# Tracing Sulfate Source and Transformation in the Groundwater of the Linhuan Coal Mining Area, Huaibei Coalfield, China

**DOI:** 10.3390/ijerph192114434

**Published:** 2022-11-04

**Authors:** Lili Cheng, Chunlu Jiang, Chang Li, Liugen Zheng

**Affiliations:** Anhui Province Engineering Laboratory for Mine Ecological Remediation, School of Resources and Environmental Engineering, Anhui University, Hefei 230601, China

**Keywords:** sulfate, sulfate isotopes, groundwater, unsaturated zone, soil profile

## Abstract

Mining activities cause surface sulfate enrichment, which has negative impacts on human health and ecosystems. These high concentrations of sulfate may enter groundwater through the unsaturated zone (UZ), threatening groundwater quality. Therefore, we combined hydrochemical and dual isotopic analyses of sulfate in surface water, soil water and groundwater with evaluations of the UZ to identify the groundwater sulfate source and transformation in the coal mining area. Soil profile samples were collected near gangue heaps (UZ−1, UZ−2) and the mean sulfate concentrations of the UZ−1 profile and UZ−2 profile were 35.4 mg/L and 69.63 mg/L, respectively. The shallow groundwater sulfate was mainly from dissolution of evaporite, sulfide oxidation and sewage. Different sulfate contaminated areas showed different characteristics of sulfate sources. The sulfate source to groundwater near the coal gangue heaps was sulfide oxidation. The groundwater sulfate near the gangue heaps and industrial park compound contamination area was mainly derived from industrial and domestic sewage and sulfide oxidation. In addition, the role of bacterial sulfate reduction (BSR) in the groundwater was not obvious. This research result is of great significance for promoting the safe mining of coal resources and sustainable utilization of groundwater in the Huaibei coal mining area and other coal mining areas in China.

## 1. Introduction

Coal is the dominant form of energy in China [1]. The Huaibei coalfields are an important coal base in China, and they have been mined for coal since the 1960s. Sulfate is a typical anion in aqueous environments and is ignored as a contaminant in groundwater. In general, the highest levels of sulfate in groundwater are from natural sources such as the dissolution of minerals (such as gypsum) and rainfall [2,3,4,5]. However, with the intensification of coal mining activities, the aqueous environment of the coalfields has been polluted due to coal gangue accumulation, mine drainage, leachate from tailings heaps, etc. Coal mining activities have become another factor in the increased sulfate load in groundwater. The potential sources of SO_4_^2−^ in coal mining areas include natural sources, such as the dissolution of gypsum, oxidation of sulfides (pyrite) and atmospheric precipitation [6,7,8], and anthropogenic sources, such as discharges of domestic and industrial wastewaters, the use of agricultural fertilizers, and coal mining activities [9,10,11]. Increases in the SO_4_^2−^ concentrations of mining-affected ecosystems can have negative environmental impacts, including acidification and the release of heavy metals and methyl mercury into aquatic systems [12,13]. The concentration of SO_4_^2−^ is limited to below 250 mg/L by the WHO and by the drinking water standards in China. High SO_4_^2−^ concentrations can cause health issues such as diarrhea and gastrointestinal disorders [14,15]. Water contamination with sulfate not only threatens human health and ecological functions, but also affects carbonate weathering and the evolution of the global carbon and nitrogen cycles [16,17,18,19]. It has been reported that the concentration of SO_4_^2−^ in the surface water of the coal mining area was high, over 250 mg/L, and sulfate contamination of the groundwater has also been noted [20,21]. For the utilization and protection of regional water resources, it is of great significance to understand the sources and transformation processes of sulfate under the effect of mining.

The dual isotopes (δ^34^S−SO_4_^2−^ and δ^18^O−SO_4_^2−^) of SO_4_^2−^ have served as powerful tracers to distinguish the sources and migration and transformation processes of sulfate along flow paths [22,23,24,25,26]. The main factors controlling the δ^34^S−SO_4_^2−^ and δ^18^O−SO_4_^2−^ values of sulfate include geological and hydrological settings, geographical location, precipitation, and biological activity (bacterial sulfate reduction, BSR). It is worth noting that BSR is the key process in the earth’s sulfur cycle causing significant isotopic fractionation and reducing the sulfate concentrations in closed systems [27,28]. The hydrogeochemical characteristics of formation water can also provide useful information about the evolution of formation water, such as the degree of water-rock interactions and the source of solutes. Therefore, using a combination of a hydrochemical technique and dual isotope approach is an effective way to elucidate the sulfate sources and transformation processes in the hydrological systems [29,30,31].

The unsaturated zone (UZ) links surface water and groundwater and plays an important role in the hydrological cycle [32,33,34]. The UZ is an important site for SO_4_^2−^ recycling and transformation and is also the main route for surface SO_4_^2−^ pollution to enter groundwater [35,36,37]. The groundwater table is low, and the UZ is shallow in the coal mining area. Due to extensive mining activities, sulfate has accumulated in the shallow UZ, posing a potential threat to the shallow groundwater resources. Previous studies have mainly focused on the identification of sulfate sources in surface water, hydrochemical characteristics of karst water, water–rock interaction mechanisms of deep aquifers, water-inrush source identification, etc. [27,38]. For example, Chen et al. [39] revealed the hydrochemical characteristics and spatial–temporal evolution mechanisms of a multiaquifer groundwater system disturbed by mining operations. Chen et al. [21] focused on the comparison of water chemistry and sulfur and oxygen isotopes between natural surface water (river water) and contaminated mine subsidence water and traced the source of sulfate in the river and subsidence water in the mining area. The above research has laid a solid foundation for this study. However, the source and transformation of sulfate in groundwater of the study area have not been systematically studied, and local investigations cannot provide sufficient information about the sulfur cycle. This study focused on the surface water, groundwater and soil water in the UZ of the Linhuan mining area in Huaibei. The main research objectives were (1) to determine the content and distribution characteristics of sulfate in the surface water, soil water and groundwater of the study area; (2) to reveal the sources of sulfate in the UZ; and (3) to determine the transformation characteristics and sources of sulfate in local groundwater. The findings provide a reference for developing effective strategies to reduce the SO_4_^2−^ inputs associated with mining activities and to determine appropriate measures to balance economic development with water quality protection in coal mining areas.

## 2. Materials and Methods

### 2.1. Study Sites

The Linhuan coal mining area, with a history of nearly 30 years of coal mining, is situated in Huaibei, Anhui Province, China. It is approximately 30.0 km east of Suzhou and 40.0 km north of Huaibei, covering a total area of approximately 1750 km^2^. The study area is subject to a semihumid climate and is in a warm belt with seasonal monsoons and four distinct seasons, and the annual average temperature is 14.5 °C. The annual average precipitation is 830 mm, most of which is concentrated in July and August. Moreover, the annual average evaporation is 1045.2 mm.

**Figure 1:**
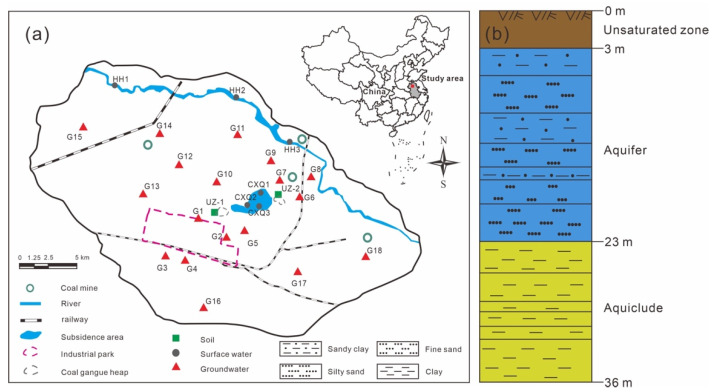
(**a**) Map of the study area. Surface water, groundwater, and soil samples are shown as gray closed circles, red closed triangles and green closed squares, respectively. The coal mining area is shown as blue open circles. (**b**) Hydrogeological cross section of the study area. The unsaturated zone, the first aquifer and the first aquiclude mainly occur at depths of 0–3 m, 3–23 m and 23–36 m in the unconsolidated Cenozoic strata.

There are 4 mines in the mining area, all of which are large underground mines. The industrial park, located in the southern part of the study area, includes coking plants, coal preparation plants, coal-fired power plants, cement plants, etc. Residential areas are present outside the industrial park and subsidence area. The constructions and mining activities at the coal mining area have already caused adverse environmental effects, such as large numbers of coal gangue heaps and large areas of water accumulation in the subsidence area. The surface water in the study area mainly consists of a river and subsidence area water. The main river is the Huihe River, which is a seasonal river flowing from northwest to southeast. The upper reaches of the Huihe River are the main source of Quaternary sediment detrital materials, and these sediments are rich in gypsum and mirabilite in parts of the upper reaches [40]. The subsidence area water is concentrated north of the Linhuan industrial park and south of the Huihe River. Two coal gangue heaps are located on the eastern and western sides of the subsidence area. The cumulative area of the subsidence area is approximately 175.46 hm^2^, with an average water depth of 3.45 m and a maximum depth of 10.0 m (Figure 1a).

The study area is a flat plain, in which the elevation ranges from +23 to +28 m and gradually decreases from northwest to southeast. The component strata (from bottom to top) consist of Ordovician, Carboniferous, Permian, Paleogene, Neogene and Quaternary strata. The coal gangue is mostly composed of sandstone, mudstone, sandy shale and trace amounts of low−S, low−pyrite coal in Carboniferous and Permian strata [41]. There are relatively thick unconsolidated Neogene and Quaternary strata in the study area, with thicknesses of 186–291 m (mean 238 m). As shown in Figure 1b, the unsaturated zone is at a depth of 0–3 m. According to the lithologic assemblages and comparisons with regional hydrogeological profiles, four aquifer groups and three aquiclude groups can be distinguished. The shallow groundwater is located in the first aquifer at the top (3–23 m depth), with a thickness of 20 m and a water table depth of 3–5 m, which is the main water source for anthropic uses and irrigation in the study area. The first aquiclude is at a depth of 23–36 m, with a thickness of 5–30 m (mean 13 m). Fine sand and silty sand with 2–3 layers of thin sandy clay are the main components of the aquifer.

### 2.2. Sampling and Sample Preparation

A sampling campaign was launched in December 2021. The samples included 6 surface water samples, 18 groundwater samples and 2 soil profiles. The distribution of sampling points was shown in Figure 1a. The depth of shallow groundwater is different in time, and the variation amplitude is generally between 1 and 3 m. Therefore, two soil profile samples (UZ−1 and UZ−2) of the unsaturated zone at a 2 m depth were collected near the coal gangue heaps 500 m from the subsidence area. We collected soil profile samples with a soil sampling drill, and 2000 g of bulk sample were collected at intervals of 0.4 m at 0–2 m. All soil samples were immediately sealed in polyethylene bags after collection, dried, crushed and passed through a 0.15 mm sieve until concentration and isotopic analyses of water-soluble sulfate.

The Huihe River and subsidence area water were selected for surface water sampling. The shallow underground water samples were drilled in the industrial park (G1–G4) and self-provided wells by residents around the mining area (G5–G18). Shallow groundwater samples were provided by residents from wells around the mining area, with depths of less than 10 m. All water samples were collected in high−density polyethylene samplers, transported to the laboratory and then filtered with 0.45 μm membranes. The samples designated for the cation test were acidified to pH < 2 by HNO_3_. Each water sample was refrigerated at 4 °C for further hydrochemical and isotopic analyses.

### 2.3. Hydrochemical and Isotopic Analyses

The basic physical and chemical parameters of the water samples, including electrical conductivity (EC), pH, dissolved oxygen (DO) and temperature (T), were measured in situ using a pH meter (WTW−PH3110, WTW, Munich, Germany) and a portable water quality meter (WTWoxi315i, WTW, Munich, Germany). The samples were analyzed for anions (SO_4_^2−^ and Cl^−^) using ion chromatography (DIONEX, model ICS−1500, Dell, Inc., TX, USA) with an analytical precision of 0.01 mg/L. To obtain the water-soluble sulfate concentration of the soil water, 50 mL of deionized water were added to 5 g of soil and extracted for 6 h [42]. The supernatant solution was filtered through 0.22 μm membranes. The SO_4_^2−^ concentration of the soil water was analyzed by ion chromatography. The anionic standard was according to Chinese Environmental Protection Standard HJ/T 84–2001. The samples were analyzed for cations (K^+^, Na^+^, Ca^2+^, Mg^2+^ and Fe) using ICP−AES (IRIS Intrepid II XSP, Thermo Fisher Scientific, Waltham, MA, USA) with 0.01 mg/L testing accuracy. The standards (GSB−1738, GSB−1736, GSB−1735 and GSB−1720) were obtained from the National Standards Center at a concentration of 1.0 mg/L. The cations were measured in accordance with the Chinese Environmental Protection Standard HJ/776−2015. The content of HCO_3_^−^ was determined by double-indicator titration. Phenolphthalein, methyl red and a standard solution of hydrochloric acid (0.05 mol·L^−1^) were prepared. The phenolphthalein indicator was added to the water sample and titrated with standard acid until colorless. The methyl red indicator was added and titrated again with standard acid until the solution turned orange-red. The content of HCO_3_^−^ was determined by two doses of standard acid. Each sample was titrated three times, and the control error was less than 5%. The mass concentration of total dissolved solids (TDS) was calculated by subtracting the 1/2HCO_3_^−^ mass concentration from the sum of the mass concentrations of the anions and cations. The saturation index of minerals in water samples was calculated using PHREEQC. In terms of data quality, to ensure accuracy, data points with an error of more than 5% were excluded from the anion-cation balance analysis.

For the δ^34^S−SO_4_^2−^ and δ^18^O−SO_4_^2−^ analyses of water samples, the filtered samples were acidified to pH < 2 by distilled 6 mol/L HCl, and the SO_4_^2−^ in the acidified samples was precipitated by the addition of 10% BaCl_2_ solutions. Then, the BaSO_4_ solutions were boiled in an electric furnace, and the precipitates were filtered through 0.22 μm membranes. The BaSO_4_ samples were ultimately obtained after the precipitates were dried in a muffle furnace at 850 °C for 2 h. For soil profile samples, 1000 g soil samples were ground into powder (100 mesh) and then extracted with 3000 mL of deionized water for 24 h. The extracted samples were filtered through 0.22 μm membranes and then treated in the same manner as the water samples described above for δ^34^S−SO_4_^2−^ and δ^18^O−SO_4_^2−^ analyses.

The sulfur and oxygen isotopes of all samples were analyzed using an elemental analyzer−stable isotopic ratio mass spectrometer (Thermo Fisher, Delta v Plus, Waltham, MA, USA). The BaSO_4_−S samples were converted to SO_2_ at 1000 °C, and the ^34^S/^32^S ratios of BaSO_4_ were measured for SO_2_ gas. BaSO_4_−O was introduced to C at temperatures between 1350 and 1500 °C and was quantitatively converted to CO. The ^18^O/^16^O ratios of BaSO_4_ were analyzed from the generated CO gas. The accuracy of sulfur and oxygen isotopic analyses was checked periodically by analyses of international reference barium sulfates (NBS127) and International Atomic Energy Agency materials (IAEA−S05, IAEA−S06). The analytical precision values of δ^34^S−SO_4_^2−^ and δ^18^O−SO_4_^2−^ were better than ±0.2% and ±0.3‰, respectively. The stable isotopic compositions of sulfur and oxygen were reported as δ values (‰), the isotopic ratio in delta relative to standards:

$$\delta_{\mathrm{sample}}(‰)=[(R_{\mathrm{sample}}{-R}_{\mathrm{standard}}{)/R}_{\mathrm{standard}}]\times 1000$$

where R is the isotopic ratio of sulfur or oxygen (^34^S/^32^S, ^18^O/^16^O) and R_sample_ and R_standard_ are the isotopic ratios of the sample and standard, respectively. The international standards for sulfur and oxygen are V−CDT and V−SMOW, respectively.

## 3. Results and Discussion

### 3.1. Hydrogeochemical Processes

**Figure 2:**
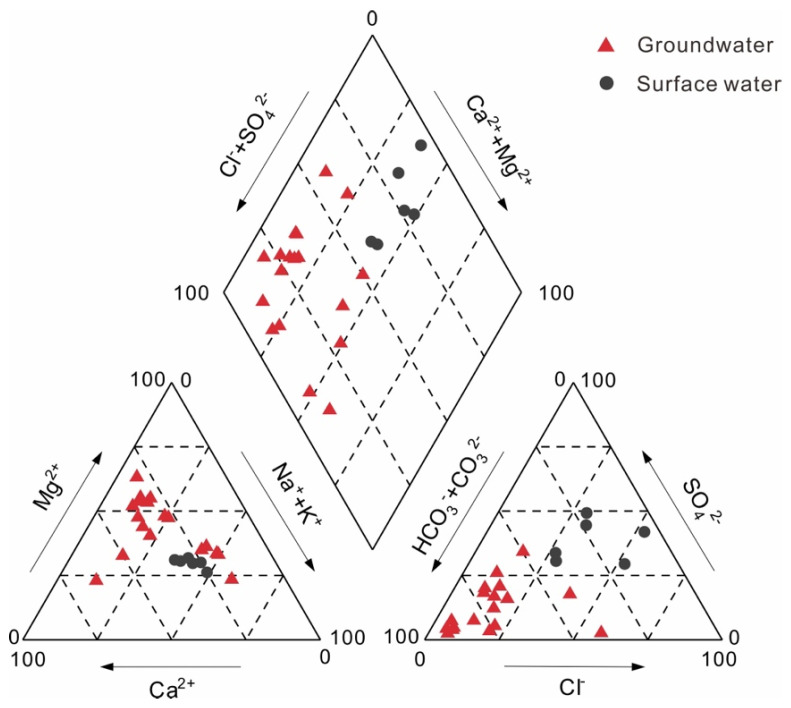
The piper diagram of surface water and groundwater samples in the mining area. The arrows point from high ion content to high ion content in water.

The hydrochemical compositions of the surface water and groundwater in the coal mining area are listed in Table 1. The surface water and groundwater were generally weakly alkaline and had low TDS and high Ec. The Huihe River water, subsidence area water and groundwater were in an aerobic environment in the catchment. The piper diagram can reflect the main ion compositions and chemical characteristics of the water. As shown in Figure 2, two hydrochemical types of water samples were classified: (a) Na·Ca−Cl·SO_4_ for surface water and (b) Ca·Mg−HCO_3_ for groundwater. Most groundwater was different from river water and subsidence area water in hydrochemical composition.

**Figure 3:**
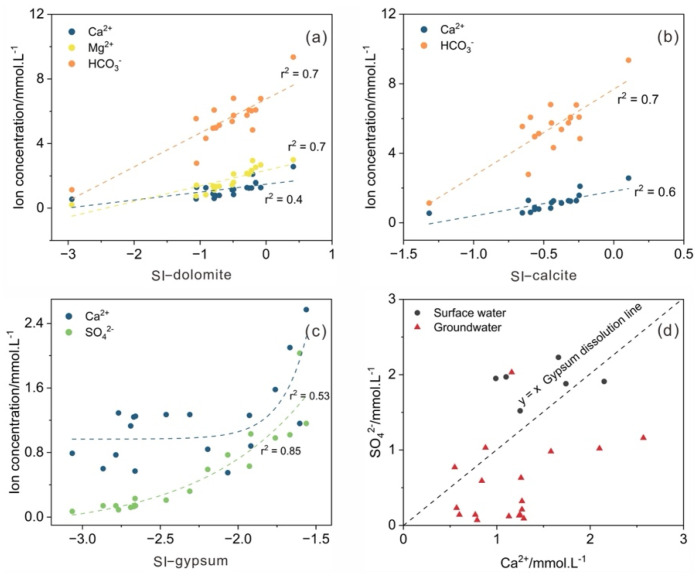
Relationship diagrams for major ions in surface water and groundwater samples: (**a**) correlations of SI−dolomite and related ions; (**b**) correlations of SI−calcite and related ions; (**c**) correlations of SI−gypsum and related ions; (**d**) scatter plot of Ca^2+^ and SO_4_^2−^.

Hydrochemical composition can provide useful information about water hydrogeochemical processes, such as the origin of solutes and the extent of water−rock interactions [43,44]. Ion ratio analysis has been widely used to analyze the hydrochemical compositions. In Figure 3a,b, the main groundwater ions were highly correlated with the carbonate SI (SI−dolomite, SI−calcite), indicating that Ca^2+^, Mg^2+^ and HCO_3_^−^ in groundwater were mainly derived from carbonate mineral dissolution. The SI−gypsum in groundwater samples was less than 0, ranging from −1.6 to −3.1 with an average of −2.3, indicating that gypsum was always dissolved along the flow path. Figure 3c showed the relationship between Ca^2+^, SO_4_^2−^ and SI−gypsum. SI−gypsum had a higher association with SO_4_^2−^ (r^2^ = 0.85) than Ca^2+^ (r^2^ = 0.5), which proved that gypsum could dissolve and release SO_4_^2−^. The weak correlation between Ca^2+^ and SI−gypsum was mainly due to the dissolution of carbonate and cation exchange. The ideal molar ratio of the major ions of gypsum (CaSO_4_·2H_2_O) should be 1. In Figure 3d, the concentration of Ca^2+^ in most groundwater samples from the Linhuan mining area was higher than that of SO_4_^2−^, which further verified that the dissolution of carbonate minerals was the dominant factor in hydrogeochemical processes in this area. The concentration of SO_4_^2−^ was higher than that of Ca^2+^ in the other samples, which may be due to the existence of cation exchange in addition to gypsum dissolution.

### 3.2. Sulfate Distributions in the Surface Water, UZ and Groundwater

**Figure 4:**
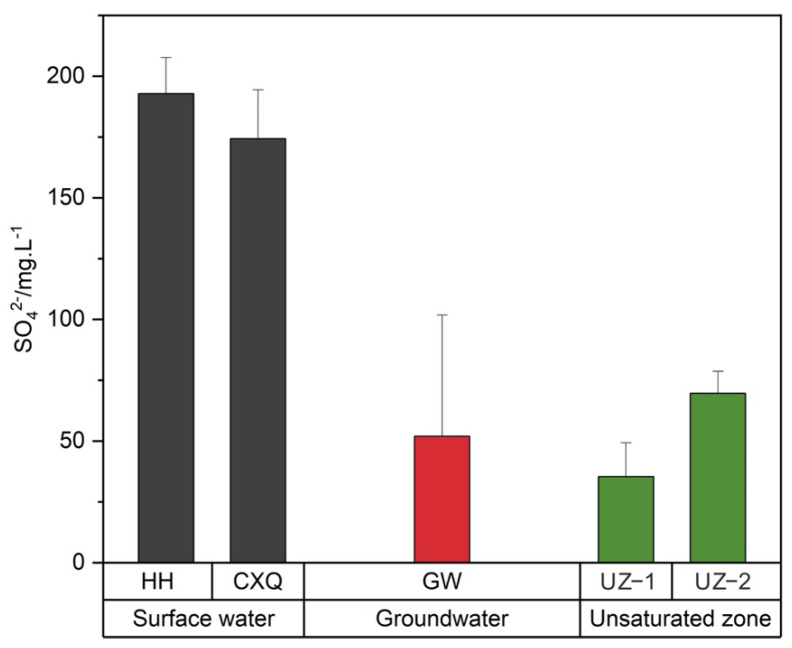
Variations in mean SO_4_^2−^ concentration in the surface water, groundwater and soil profile samples in the coal mining area.

The SO_4_^2−^ concentrations of surface water, soil profiles, and groundwater are shown in Table 2 and Figure 4. Chen et al. [45] found that the mean SO_4_^2−^ concentration was 386.7 mg/L in the rivers of the Huaibei coalfield between 2015 and 2017. In contrast, the mean SO_4_^2−^ concentration in the surface water was slightly lower at the present site, and the temporal variation in SO_4_^2−^ in the surface water was large. The sulfate concentration of the unconsolidated aquifer varied only slightly [39]. The sulfate concentrations of G2 (98.85 mg/L), G3 (97.67 mg/L) and G13 (111.53 mg/L) were higher than those of other groundwater samples, as these samples were affected by industrial wastewater and domestic sewage from the industrial park. Sampling point G6 (195.13 mg/L) had the highest sulfate concentration, mainly due to the weathering and leaching of the gangue heaps and dissolution of evaporite. The SO_4_^2−^ concentrations of the soil profiles (UZ−1 and UZ−2) increased slightly with soil depth to reach maximum values at 200 cm (Figure 4). The uppermost part of the Cenozoic section is recharged directly by atmospheric precipitation. In addition, the upper stratum has favorable factors, such as good sorting and small compactness, so the runoff conditions are good, and elements can easily migrate. Leaching results in a higher sulfate concentration in the UZ with increasing depth. The SO_4_^2−^ concentration (water−soluble sulfate and adsorbed sulfate) of the soil solution in the Linhuan mining area ranged from 11.08 to 59.18 mg/L (mean 25.38 mg/L) in 2019 [46]. Compared with the previous data, there was a distinct SO_4_^2−^ enrichment phenomenon in the UZ−1 profile and UZ−2 profile. The SO_4_^2−^ concentration of the UZ can be affected by seasonal variations, fertilization and organic sulfur mineralization, as well as by atmospheric deposition and coal gangue accumulation [47].

### 3.3. Sulfate Sources in the Groundwater

In coal mining areas, sulfate can originate from various potential sources, including natural sources, such as atmospheric precipitation, dissolution of evaporite and oxidation of sulfide, and anthropogenic sources, such as sewage and mine drainage [48,49,50,51,52,53,54]. The δ^34^S−SO_4_^2−^ and δ^18^O−SO_4_^2−^ values of the surface water ranged from +14.5 to +20.2‰ (mean +18.0‰) and +9.3 to +11.2‰ (mean +10.3‰), respectively. The δ^34^S−SO_4_^2−^ and δ^18^O−SO_4_^2−^ values of the groundwater ranged from +7.5 to +21.8‰ (mean +11.7‰) and +5.3 to +15.7‰ (mean +10.6‰), respectively (Table 2).

**Figure 5:**
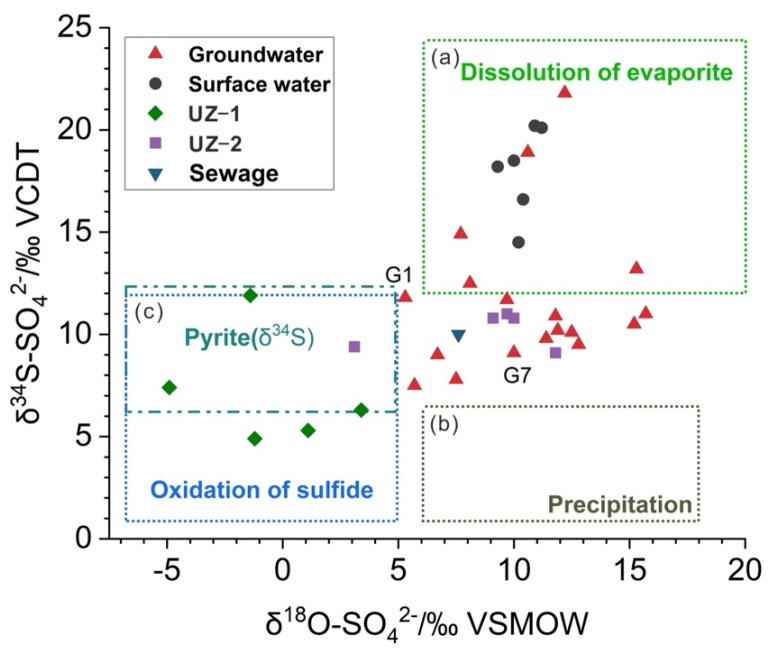
Compositions of δ^34^S−SO_4_^2−^ and δ^18^O−SO_4_^2−^ in the study area. The typical values of δ^34^S−SO_4_^2−^ and δ^18^O−SO_4_^2−^ for (**a**) dissolution of evaporites ranged from δ^34^S−SO_4_^2−^ = +12‰ to +35‰, δ^18^O−SO_4_^2−^ = +6‰ to +20‰; (**b**) atmospheric precipitation ranged from δ^34^S−SO_4_^2−^ = −5‰ to +6‰, δ^18^O−SO_4_^2−^ = +6‰ to +18‰; and (**c**) oxidation of sulfide ranged from δ^34^S−SO_4_^2−^ = −18‰ to +12‰, δ^18^O−SO_4_^2−^ = −5‰ to +5‰ [21,56,57].

Atmospheric precipitation is the recharge source for surface water and groundwater in the study area. Atmospheric precipitation usually contains a low concentration of SO_4_^2−^ that can be transported to surface water and aquifers during recharge and runoff processes [55]. The typical δ^34^S−SO_4_^2−^ and δ^18^O−SO_4_^2−^ values in precipitation ranged from −5‰ to +6‰ and +6‰ to +18‰, respectively [56]. The δ^34^S−SO_4_^2−^ and δ^18^O−SO_4_^2−^ values of precipitation in the study area ranged from +6.3 to +8.7‰ (mean +7.2‰) and from +9.2 to +11.5‰ (mean +10.2‰), respectively [40], which were essentially consistent with the typical results of Li et al. [56]. As shown in Figure 5, the δ^34^S−SO_4_^2−^ and δ^18^O−SO_4_^2−^ values of the surface water and groundwater samples did not fall within the range of atmospheric precipitation. Additionally, the SO_4_^2−^ concentration in the precipitation (13.85 mg/L) was much lower than the mean SO_4_^2−^ concentration of the surface water (183.5 mg/L) and groundwater (50.28 mg/L). Consequently, sulfate in atmospheric precipitation cannot be the primary source that provided sulfate with low δ^34^S values to the surface water and groundwater.

Sewage is another important source for sulfate contamination in the local groundwater [58,59]. The coal mining area covers town and suburban areas. Most of the groundwater samples were collected from residential wells in villages, and a few were taken from wells drilled near industrial park areas. Sewage (e.g., domestic wastewater and manure) from these wells was not systematically treated and therefore continuously infiltrated into shallow aquifers through Cenozoic faults and unsaturated zones. Zhang et al. [8] collected and measured sewage in the North China Plain, and the mean values of δ^34^S−SO_4_^2−^ and δ^18^O−SO_4_^2−^ were 10‰ and 7.6‰, respectively. The δ^34^S−SO_4_^2−^ and δ^18^O−SO_4_^2−^ values of some of the groundwater samples were close to the range of sewage (Figure 5), which also confirmed the important contribution of sewage to groundwater sulfate.

The dissolution of evaporite can affect SO_4_^2−^ concentrations in surface water and groundwater and enrich isotopic compositions [60,61]. Previous studies have shown that a large number of gypsums were present in the strata of the study area [41]. The coal mining activities damaged the bottom plate of aquifers, which led to linkages between aquifer systems and decreases in the water levels. The above process may enhance the leaching of gypsum mineral in the unsaturated zone during the recharge process, resulting in an increase in SO_4_^2−^ content in the local groundwater and surface water. As seen in Figure 4c, the SI−gypsum was less than 0 and linearly related to the sulfate content of groundwater. Gypsum was always in a dissolved condition and released SO_4_^2−^. The typical ranges of δ^34^S−SO_4_^2−^ and δ^18^O−SO_4_^2−^ for the dissolution of evaporites are between +12 and +35‰ and +6 and +20‰, respectively [56]. Figure 5 showed that all surface water samples had high δ^34^S−SO_4_^2−^ and δ^18^O−SO_4_^2−^ values, which were within the range for the dissolution of evaporite. Consequently, evaporite dissolution contributed greatly to the SO_4_^2−^ present in the surface water, which was consistent with the results of Chen et al. [21] in the study area. Evaporite dissolution also contributed to sulfate in the groundwater. The δ^34^S−SO_4_^2−^ and δ^18^O−SO_4_^2−^ values of approximately 28% for the groundwater samples were within the range (Figure 5), and the δ^34^S−SO_4_^2−^ and δ^18^O−SO_4_^2−^ values of the remaining groundwater samples were lower than those from the dissolution of evaporites. The evaporite dissolution contribution to surface water sulfate was significantly higher than that to groundwater.

### 3.4. Sulfate Sources in the UZ

**Figure 6:**
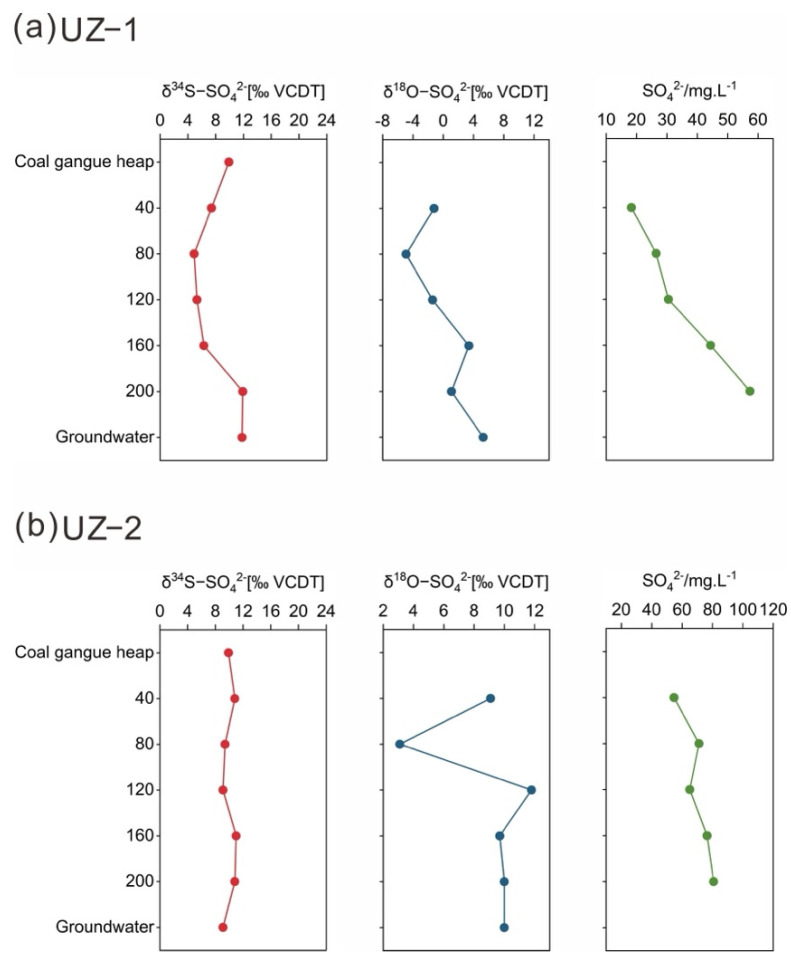
(**a**) The SO_4_^2−^concentration and isotopic data of the water−soluble sulfates (δ^34^S−SO_4_^2−^ and δ^18^O−SO_4_^2−^) for samples taken from coal gangue, soil profiles (UZ−1) and groundwater (G1); (**b**) The SO_4_^2−^concentration and isotopic data of the water−soluble sulfates (δ^34^S−SO_4_^2−^ and δ^18^O−SO_4_^2−^) for samples taken from coal gangue, soil profiles (UZ−2) and groundwater (G7).

Mining activities have resulted in large quantities of coal gangue and large areas of subsidence area water. Sulfate from the sulfide oxidation of coal gangue and subsidence area water may penetrate into groundwater through the unsaturated zone. The sulfide minerals in coal gangue are mainly found in the form of pyrite [62,63]. The environment caused by the damage of strata by mining activities is favorable for the oxidation of sulfides (pyrite) in coal gangue. Sulfide oxidation usually results in minimal S isotopic fractionation [64,65], basically preserving the original isotopic composition of the sulfide minerals. However, the oxygen in water and the atmosphere participates in sulfide oxidation in different proportions, resulting in the extremely complex behavior of δ^18^O−SO_4_^2−^ [66]. In Figure 5, the δ^34^S−SO_4_^2−^ values of most UZ samples were close to the δ^34^S−SO_4_^2−^ range (+6.3‰~+13.5‰, mean 9.9‰) of the precursor sulfide minerals (pyrite) in the coal mine area, as reported by Chen et al. [21], indicating that the δ^34^S−SO_4_^2−^ signatures resulted from the oxidation of sulfide. The mean pyrite δ^34^S−SO_4_^2−^ value (9.9‰) of the gangue samples collected in the Linhuan mining area by Chen et al. [21] and δ^34^S−SO_4_^2-^ and δ^18^O−SO_4_^2−-^ values of the soil profiles (UZ−1 and UZ−2) collected near the gangue heaps and groundwater sampling points (G1 and G7) closest to the profiles were used to reveal the migration process of sulfate in the UZ and sulfate sources in the groundwater (Figure 6). As shown in Figure 6b, the δ^34^S−SO_4_^2−^ values of the UZ−2 profile and groundwater sampling point G7 were very similar to the average δ^34^S−SO_4_^2−^ value of pyrite in the coal gangue. Meanwhile, the δ^34^S−SO_4_^2−^ values formed a narrow range from +9.1‰ to +11.0‰, and the SO_4_^2−^ content showed an increasing trend with increasing depth of the profile. Figure 6a shows that the variation characteristics of δ^34^S−SO_4_^2−^ values in the UZ−1 profile at depths of 40−160 cm were similar to those of the UZ−2 profile. This phenomenon in the UZ profiles indicated that oxidation of pyrite occurred in the coal gangue and that the resulting SO_4_^2−^ vertically migrated in the UZ. Yang et al. [46] found that the soil pH value of the subsidence area in Linhuan was generally low, and most of it was under acidic conditions. The soil near the gangue heaps showed higher levels of organic matter and sulfate content than the soil far away from the gangue heaps. Jiang et al. [67] also found that the concentrations of Fe ions in the shallow groundwater near gangue heaps in the mining area were higher than those in other areas. The higher Fe level may be due to the pyrite oxidation of the coal gangue. These studies further indicated that pyrite oxidation was the source of sulfate in groundwater near gangue heaps.

At the depth of 160–200 cm, the δ^34^S−SO_4_^2−^ values in the UZ−1 profile increased from +6.3‰ to +11.9‰, and the δ^34^S−SO_4_^2−^ value of groundwater sampling site G1 was close to the δ^34^S−SO_4_^2−^ in the UZ−1 profile (Figure 6a), which may be due to the mixing of sulfate from pyrite oxidation with sulfate from sewage. The mean value of δ^34^S−SO_4_^2−^ and δ^18^O−SO_4_^2−^ in sewage in the North China Plain was 10‰ and 7.6‰, respectively [8]. The mean value of δ^34^S−SO_4_^2−^ in sewage was higher than that of the dissolved sulfate in the UZ−1 profile (+7.2‰). The mixing of sewage with high δ^34^S−SO_4_^2−^ values increased the δ^34^S−SO_4_^2−^ values at the bottom of the UZ−1 profile (160–200 cm). Groundwater sampling point G1 was taken from wells drilled near the industrial park. The concentrations of Cd in the shallow groundwater near the industrial park were higher than those in other areas [67], indicating that the groundwater was contaminated with industrial and domestic sewage. The δ^34^S−SO_4_^2−^ and δ^18^O−SO_4_^2−^ values of the subsidence area water (CXQ1−CXQ3) ranged from +16.6‰ to +20.2‰ and +10.4‰ to +11.2‰, respectively, and the mean sulfate concentration was 174.30 mg/L. The δ^34^S−SO_4_^2−^ and δ^18^O−SO_4_^2−^ values of the subsidence area water were within the range of gypsum dissolution (Figure 5). The high concentration of SO_4_^2−^ in the subsidence area was mainly from gypsum dissolution, which was supported by Chen et al. [21]. The bottom of the subsidence area water and the Huihe River are composed of clay, sandy clay and silty clay with weak water permeability [68]. Therefore, the subsidence water had little effect on the shallow groundwater.

### 3.5. Biogeochemical Processes of Sulfate

BSR is a biogeochemical process. Under anaerobic conditions, sulfate is decomposed by the BSR reaction and is eventually reduced to sulfide as the end product [69,70]. The chemical equation is as follows:

$$SO_{4}^{2-}+2CH_{2}O=H_{2}S+2HCO_{3}^{-}$$

Certain transitions occur in the sulfur and oxygen isotopic ratios of sulfate during the BSR process. In the early stages of BSR, the values of both δ^34^S−SO_4_^2−^and δ^18^O−SO_4_^2−^ change rapidly. However, with the consumption of sulfate, the δ^34^S−SO_4_^2−^ value continues to increase, and the δ^18^O−SO_4_^2−^ value of the remaining sulfate may approach a constant value, resulting in the enrichment of ^34^S in the remaining sulfate [71,72,73,74].

**Figure 7:**
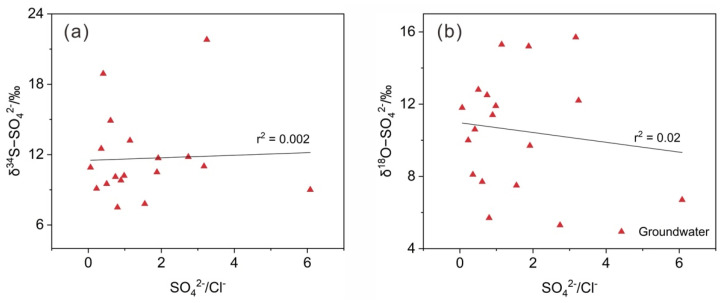
Relationships among δ^34^S−SO_4_^2−^ and δ^18^O−SO_4_^2−^ and SO_4_^2−^/Cl^−^ of the groundwater in the coal mining area. (**a**) correlations of SO_4_^2−^/Cl^−^ and δ^34^S−SO_4_^2−^; (**b**) correlations of SO_4_^2−^/Cl^−^ and δ^18^O−SO_4_^2−^.

As seen in Figure 7, no significant trend was observed between the δ^34^S−SO_4_^2−^ and δ^18^O−SO_4_^2−^ values and SO_4_^2−^/Cl^−^ in the groundwater. Additionally, the mean value of DO in the groundwater was 4.35 mg/L, which also verified that the redox conditions were insufficient to achieve the reduction of SO_4_^2−^ [75]. All these results confirmed that BSR did not significantly change the original isotopic composition of the sulfate sources in the groundwater.

## 4. Conclusions

A tracer study using chemical and isotopic fingerprints was conducted to illuminate the source and transformation of sulfate in groundwater in the coal mining area.

(1) The results of sulfate isotopes showed that SO_4_^2−^ in the shallow groundwater was mainly derived from the natural dissolution of evaporite, oxidation of sulfide and sewage.
(2) The oxidation of sulfide was sulfate source to groundwater near the gangue heaps. The groundwater sulfate near the gangue heaps and industrial park compound contamination area was mainly affected by oxidation of sulfide and industrial and domestic sewage.
(3) During the process, BSR did not affect the sulfate isotopic composition of groundwater.

Sewage and coal gangue, which are produced by human activities and mining, respectively, are important factors leading to SO_4_^2−^ contamination in the regional groundwater. Therefore, the local government should improve domestic sewage treatment infrastructure and treat coal gangue heaps in a timely manner to prevent SO_4_^2−^ from polluting groundwater and surface water.

## Figures and Tables

**Table 1:** Hydrochemical composition and saturation index of surface water and groundwater samples in the coal mining distric.

Type		pH	Ec	DO	K^+^	Na^+^	Ca^2+^	Mg^2+^	Fe	Cl^−^	SO_4_^2−^	HCO_3_^−^	TDS	SI−c *	SI−d *	SI−g *
			us/cm	mg/L												
	Min	7.64	1238.0	9.21	2.83	79.01	39.45	24.71	0.18	83.05	145.87	33.53	475.58	−1.30	−2.48	−1.64
	Max	7.86	1928.0	11.26	13.37	108.41	85.82	47.88	23.30	239.08	213.68	304.43	735.35	−0.33	−0.48	−1.40
Surface water	Mean	7.74	1582.67	10.54	5.84	92.08	59.21	36.86	6.20	138.01	183.54	154.01	592.55	−0.78	−1.40	−1.52
	SD	0.07	297.40	0.76	3.45	10.01	16.17	8.36	8.89	59.34	19.94	86.09	96.42	0.32	0.65	0.10
	CV	0.01	0.19	0.07	0.59	0.11	0.27	0.23	1.43	0.43	0.11	0.56	0.16	−0.41	−0.46	−0.07
	Min	7.76	590.00	1.90	0.31	4.17	22.14	4.80	0.02	8.95	6.61	68.93	93.04	−1.32	−2.94	−3.07
	Max	8.31	1604.00	7.52	2.05	235.75	102.73	71.79	31.18	159.83	195.13	570.35	701.62	0.11	0.41	−1.56
Groundwater	Mean	7.94	887.94	4.35	0.71	44.46	46.94	41.55	3.08	47.69	52.33	331.12	399.24	−0.45	−0.63	−2.31
	SD	0.14	292.68	1.73	0.53	31.82	20.20	18.35	8.48	41.59	49.76	100.15	141.73	0.28	0.67	0.47
	CV	0.02	0.33	0.40	0.74	0.72	0.43	0.44	2.75	0.87	1.00	0.30	0.36	−0.61	−1.07	−0.20

* SI−c, SI−d, and SI−g represent the saturation index (SI) of calcite, dolomite, and gypsum, respectively.

**Table 2:** Sulfate concentration and sulfur and oxygen isotopic composition of sulfate in the surface water, soil profiles (UZ−1 and UZ−2) and groundwater.

Type	Sample No./Depth (m)	δ34S−SO_4_^2−^ (‰)	δ18O−SO_4_^2−^ (‰)	SO_4_^2−^ (mg/L)
	HH1	14.5	10.2	213.7
	HH2	18.5	10	180.8
	HH3	18.2	9.3	183.8
Surface water	CXQ1	16.6	10.4	145.9
	CXQ2	20.1	11.2	189.4
	CXQ3	20.2	10.9	187.7
	Mean ± SD	18.0 ± 2.0	10.3 ± 0.6	183.5 ± 19.9
	CV	0.1	0.1	0.1
	0–40	7.4	−1.2	18.3
	40–80	4.9	−4.9	26.4
	80–120	5.3	−1.4	30.5
UZ−1	120–160	6.3	3.4	44.4
	160–200	11.9	1.1	57.4
	Mean ± SD	7.2 ± 2.5	−0.6 ± 2.8	35.4 ± 13.9
	CV	0.4	−4.6	0.4
	0–40	10.8	9.1	54.7
	40–80	9.4	3.1	71.2
	80–120	9.1	11.8	65.1
UZ−2	120–160	11.0	9.7	76.5
	160–200	10.8	10.0	80.8
	Mean ± SD	10.2 ± 0.8	8.7 ± 3.0	69.6 ± 9.1
	CV	0.1	0.3	0.4
	G1	11.8	5.3	60.0
	G2	11	15.7	98.9
	G3	14.9	7.7	97.7
	G4	9	6.7	13.2
	G5	10.2	11.9	56.7
	G6	21.8	12.2	195.1
	G7	9.1	10.0	94.3
	G8	12.5	8.1	20.3
	G9	11.7	9.7	12.8
Groundwater	G10	10.1	12.5	11.73
	G11	18.9	10.6	74.2
	G12	9.8	11.4	13.0
	G13	7.8	7.5	111.5
	G14	10.9	11.8	9.0
	G15	7.5	5.7	30.7
	G16	13.2	15.3	13.9
	G17	9.5	12.8	6.6
	G18	10.5	15.2	22.4
	Mean ± SD	11.7 ± 3.6	10.6 ± 3.1	52 ± 49.8
	CV	0.3	0.3	1.0

## Data Availability

Not applicable.
